# Application values of prenatal screening and non-invasive gene sequencing in fetal birth defects

**DOI:** 10.12669/pjms.36.7.2290

**Published:** 2020

**Authors:** Jingjing Bu, Pan Jiang, Xiaoli Cui, Hongyan Zhou, Fengxia Han

**Affiliations:** 1 Jingjing Bu Central laboratory, Binzhou People’s Hospital, Shandong, 256600, China; 2 Pan Jiang Central laboratory, Binzhou People’s Hospital, Shandong, 256600, China; 3 Xiaoli Cui Central laboratory, Binzhou People’s Hospital, Shandong, 256600, China; 4 Hongyan Zhou Clinical Laboratory, Binzhou People’s Hospital, Shandong, 256600, China; 5 Fengxia Han Clinical Laboratory, Binzhou People’s Hospital, Shandong, 256600, China

**Keywords:** Prenatal screening, Non-invasive gene sequencing, Fetal birth defects, Pregnancy outcome

## Abstract

**Objective:**

To investigate effects of prenatal screening and non-invasive gene sequencing on the clinical diagnosis of fetal birth defects and the outcome of pregnancy.

**Methods:**

Totally 2520 pregnant women who received prenatal screening in our hospital were selected as the research subjects. The high-risk pregnant women were further tested by the non-invasive gene sequencing technology. Pregnant women with positive results were diagnosed by amniocentesis and fetal chromosome karyotype analysis, and the pregnancy outcome was followed up for one year.

**Results:**

870 out of the 2520 pregnant women was tested by non-invasive gene sequencing technology; 26 of the 870 women was 13-trisomy-positive and was diagnosed by amniocentesis and fetal chromosome karyotype analysis, 22 of which was diagnosed as 47, XN, +13 and four of which was normal; the diagnosis accuracy of non-invasive prenatal testing (NIPT) was 84.6%. 18 out of the 22 confirmed cases underwent abortion, three cases had termination of embryonic development, and one case had postnatal anomaly. Thirty four out of the 2520 pregnant women was 18-trisomy-positive and was diagnosed by amniocentesis and fetal chromosome karyotype analysis, 31 of which was diagnosed as 47, XN, +18 and three cases were normal; the diagnosis accuracy of NIPT was 91.2%. 29 out of the 31 confirmed cases underwent abortion and two cases had termination of embryonic development. Forty out of the 2520 pregnant women was 21-trisomy-positive and was diagnosed by amniocentesis and fetal chromosome karyotype analysis, 39 of which was diagnosed as 47, XN, +21 and one case was normal; the diagnosis accuracy of NIPT was 97.5%. Thirty four out of the 39 confirmed cases underwent abortion, three cases had termination of embryonic development, and two cases had postnatal anomaly. Twenty eight cases were tested as sex chromosome-positive and were diagnosed by amniocentesis and fetal chromosome karyotype analysis, 25 out of which was diagnosed as abnormal and three cases were normal; the diagnosis accuracy of NIPT was 89.3%. 24 out of the 25 confirmed cases underwent abortion, and one case had termination of embryonic development.

**Conclusion:**

Prenatal screening and non-invasive gene sequencing technology have a high accuracy in the diagnosis of fetal birth defects, which can reduce the maternal abortion injury as much as possible and relieve the psychological pressure. The promotion of the mode can be strengthened in clinics.

## INTRODUCTION

Birth defects mainly refer to congenital diseases such as body structure, function or metabolism abnormality before birth, which is the main cause of infant death and congenital disability in clinic.^1^,^2^ According to statistics, the current incidence of birth defects in China is about 4% ~ 6%. In recent years, due to the increase of the birth rate of the second child, the incidence of birth defects has a significant increase trend.^3^ Chromosomal disease is the main disease of fetal birth defects, including 13-trisomy syndrome, 18-trisomy syndrome and 21-trisomy syndrome.^4^,^5^

At present, there is no effective treatment for this kind of disease, which can only be diagnosed and prevented in time by prenatal screening and non-invasive gene sequencing technology.^6^

Noninvasive DNA detection is to sequence the free DNA fragments in maternal peripheral plasma by using a new generation of DNA sequencing technology by extracting the venous blood of pregnant women and analyze the biological information of the sequencing results, from which fetal genetic information can be obtained; the genome coverage rate is as high as 93%.

Ji pointed out that non-invasive gene sequencing technology as the main detection technology could improve the efficiency of prenatal examination in the development of prenatal examination of pregnant women.^7^ He found that the non-invasive gene sequencing technology could analyze the related diseases according to the DNA gene sequence fragment provided by pregnant women, scientifically identify the growth status of the fetus in the maternity examination, and analyze the fetal trisomy 21 syndrome, trisomy 18 syndrome, monosomy 18 syndrome, trisomy 13 syndrome, and X chromosome and Y chromosome abnormalities, i.e., the technology could meet the practical needs of fetal birth defects detection. Feng et al. found that the most significant feature of non-invasive gene sequencing technology was that the harm to pregnant women was reduced to the minimum or even zero and pregnant women had no pain in the detection process.^8^ Based on this, the authors selected 2520 pregnant women in the second trimester who underwent prenatal screening in our hospital in the past one year as the research subjects, carried out non-invasive gene sequencing technology and amniocentesis chromosome karyotype diagnosis for those with abnormal results, and analyzed the clinical effect of the diagnosis results on the prevention of fetal birth defects, as well as the impact on pregnancy outcome.

## METHODS

Two thousand two hundred fifty pregnant women in the second trimester who underwent prenatal screening from April 2018 to May 2019 were selected as the main subjects of study. All pregnant women were singleton, have been pregnant for 17-20 weeks, have gave birth for one to three times (average (2.3±0.9) times), and aged 18-47 years old (average (28.17±2.35) years old). The detection subjects included pregnant women who showed with high risk, critical risk and single value abnormality in Down’s syndrome screening, showed nuchal translucency (NT) thickening in ultrasonic examination, were elderly, have exceeded the gestational age, or were required to receive examination. Family members of the fetus were informed with the study protocol and were willing to take relevant risks. They have signed the informed consent for the study, and the study was approved by the medical ethics committee (Ref.No# 161, Dated January 14, 2020).

### Methods

Prenatal screening: 2-3 mL of fasting venous blood was extracted from each pregnant woman. The alpha fetoprotein (AFP), free estriol (uE3) and free-β human chorionic gonadotrophin (free-βHCG) in the serum was screened using time-resolution fluorescence immunoassay analyzer (Nanjing Lansion Biotechnology Co., Ltd.; model type: LS-2150). The risk values of fetal Patau syndrome (T13), Edwards’s syndrome (T18) and Down’s syndrome (T21) were evaluated by Multiaale software combined with the basic information of pregnant women, such as weight, gestational age and age.

### Noninvasive gene sequencing technology (NIPT)

15 mL of fasting venous blood was taken from each woman and tested by a third-party sequencing company cooperating with our hospital. After separating the plasma, the free DNA fragments (including fetal free DNA) in the maternal peripheral plasma were sequenced by a new generation of DNA sequencing technology, and the sequencing results were analyzed for biological information, from which the genetic information of the fetus could be obtained, so as to judge whether the fetus was suffering from chromosomal diseases.

### Amniocentesis and karyotype analysis

before amniotic fluid extraction, the pregnant women emptied their bladder and lay on their back at a designated position. The deepest part of amniotic fluid was identified through B-ultrasound and marked. After strict disinfection, a lumbar puncture needle was slowly punctured into the marked part and slowly withdrawn after reaching the amniotic cavity of the uterus. Then two mL of amniotic fluid which was extracted for the first time was discarded, and then 20 mL of amniotic fluid was collected by two tubes, 20 mL each tube. The amniotic fluid was centrifuged, the supernatant was discarded, the remaining amniotic fluid cells were cultured in vitro for preparation of chromosomes, and then the full-automatic intelligent chromosome karyotype analysis system (IMSTAR company, France; model type: Meta Scan Karyotyping System) was used for karyotype analysis.

### Determination criteria

According to the medical authentication, primiparas over 35 years old were pregnant woman at an elder age, and old age is one of the high-risk factors. The risk values of chromosomal diseases are divided into low risk value, critical risk value and high risk value. 13-trisomy below 1/150 is the low-risk value, 1/150~1/1000 is the critical risk value, 1/150 or higher is the high-risk value, i.e., positive. 18-trisomy below 1/350 is the low-risk value, 1/350~1/1000 is the critical risk value, 1/350 or higher is the high-risk value, i.e., positive. 21-trisomy below 1/270 is the low-risk value, 1/270~1/500 is the critical risk value, and 1/270 or higher is the high risk value, i.e., positive. Critical and high-risk pregnant women and pregnant women at an elder age were tested by non-invasive gene sequencing technology.

## RESULTS

Two thousand two hundred fifty pregnant women underwent prenatal screening, and pregnant women at an elder age accounted for 13.45% (339/2520). The prenatal screening found that pregnant women with low risk value accounted for 65.48% (1650/2520), pregnant women with critical risk value accounted for 15.04% (379/2520), and pregnant women with high risk value accounted for 6.03% (152/2520).

The detection results of the non-invasive gene sequencing technology showed that the prevalence rate was 14.7% (128/870). Among the 379 pregnant women with critical-risk value, one case was 13-trisomy syndrome-positive, one case was 18-trisomy syndrome-positive, four cases were 21-trisomy syndrome-positive, and two cases were sex chromosome syndrome-positive. Among 152 pregnant women with high risk value, 24 cases were positive for 13-trisomy syndrome, 28 cases were 18-trisomy syndrome-positive, 26 cases were 21-trisomy syndrome-positive, and 22 cases were sex chromosome syndrome-positive. Among the pregnant women at an elder age, one case had 13-trisomy syndrome-positive, five cases were 18-trisomy syndrome-positive, ten cases were positive 21-trisomy syndrome-positive, and three cases were sex chromosome syndrome-positive (Table-I).

**Table-I:** Detection results of non-invasive gene sequencing technology [n (%)].

Types of risks for pregnant women	Critical risk	High risk	Elder age	Total
Number of cases	379	152	339	870
13-trisomy-positive	1(0.26)	24(15.79)	1(0.29)	26(2.99)
18-trisomy-positive	1 (0.26)	28(18.42)	5(1.47)	34(3.91)
21-trisomy-positive	3(0.79)	26(17.11)	11(3.24)	40(4.60)
Sex chromosome-positive	2(0.53)	22(14.47)	4(1.18)	28(3.22)

The results of amniocentesis and karyotype analysis showed that the prevalence rate was 13.4% (117/870). Among twenty-six pregnant women who showed a positive NIPT result for 13-trisomy, 22 of them were confirmed as 47, XN, +13 by amniocentesis and karyotype analysis, and four of them were normal. Among thirty-four pregnant women who showed a positive NIPT result for 18-trisomy, 31 of them were confirmed as 47, XN, +18 by amniocentesis and karyotype analysis, and three of them were normal. Among forty pregnant women who showed a positive NIPT result for 21-trisomy, nine cases were confirmed as 47, XN, +21 by amniocentesis and karyotype analysis, and one case was normal. Among twenty-eight pregnant women who showed a positive NIPT result for sex chromosome, 25 cases were confirmed as abnormal by amniocentesis and karyotype analysis, and three cases were normal (Table-II).

**Table-II:** Comparison between NIPT results and results of amniocentesis and karyotype analysis [n (%)].

NIPT results	Number of cases	Number of confirmed cases	Diagnosis accuracy of NIPT
13-trisomy-positive	26	22	84.62
18-trisomy-positive	34	31	91.18
21-trisomy-positive	40	39	97.50
Sex chromosome-positive	28	25	89.29

Total	128	117	/

### Pregnancy Outcome

After the diagnosis by amniocentesis and karyotype analysis, 18 out of 22 pregnant women with 13-trisomy syndrome underwent abortion, three women selected termination of fetal development, and one case had abnormal fetus after birth; 29 out of 31 pregnant women with 18-trisomy syndrome underwent abortion, and two women selected termination of fetal development; 34 out of 39 pregnant women with 21-trisomy syndrome underwent abortion, three women selected termination of fetal development; 24 out of the 25 pregnant women with sex chromosome syndrome underwent abortion, and one women selected termination of fetal development (Table-III).

**Table-III:** Pregnancy outcome [n (%)].

Diagnosis type of amniocentesis and karyotype analysis	Number of cases	Abortion	Termination of fetal development	Fetal normal after birth	Fetal abnormal after birth
13-trisomy-positive	22	18(81.82)	3(13.64)	0(0)	1(4.54)
18-trisomy-positive	31	29(93.55)	2(6.45)	0(0)	0(0)
21-trisomy-positive	39	34(87.18)	3(7.69)	0(0)	2(5.13)
Sex chromosome-positive	25	24(96)	1(4)	0(0)	0(0)

Total	117	105(89.74)	9(7.69)	0(0)	3(2.57)

## DISCUSSION

In China, birth defects caused by chromosomal diseases occur every year, and the incidence is increasing year by year.^9^ The most common prenatal screening in clinic is to extract AFP, uE3 and free-βHCG from the serum of pregnant women, calculate the risk value of chromosomal abnormality with Multiaale software, and screen out the critical high-risk group or high-risk group that may have some kind of chromosomal disease, so as to improve the accuracy of detection.^10^,^11^

Traditional prenatal diagnosis techniques include amniocentesis, chorionic villus biopsy, fetal umbilical vein puncture and fetal tissue biopsy, but it can cause bleeding, with 0.5%-1% risk of complications such as infection and abortion.^12^ It brings great psychological pressure to pregnant women and their families and greatly reduces the compliance of prenatal diagnosis.

In order to plan the work of maternity examination for pregnant women better, people are constantly changing the technology of maternity examination in the development of clinical medicine. The non-invasive gene sequencing technology, as the main technology in the prenatal examination of pregnant women, is a new form of technology practice formed in the development of existing medicine. Non-invasive gene sequencing technology has strong practicality in prenatal examination of pregnant women as it can detect various diseases and make scientific judgment on fetal development.

The results showed that there were 1650 low-risk pregnant women, 379 critical-risk pregnant women, 152 high-risk pregnant women and 339 pregnant women at an elderly age, and 870 pregnant women might have abnormal factors and underwent non-invasive gene sequencing. Among the 870 pregnant women tested by NIPT, 26 cases were 13-trisomy-positive, 34 cases were 18-trisomy-positive, 40 cases were 21-trisomy-positive, and 28 were sex chromosome positive, which showed that NIPT had a good diagnostic performance for fetal chromosomal diseases and could effectively improve the diagnostic accuracy of prenatal screening, and the conclusion was in line with a previous research.^13^ After that, the above-mentioned patients were diagnosed by amniocentesis and karyotype analysis. The results showed that the diagnosis rate was kept above 80%, indicating that the detection accuracy of the gene sequencing technology was not 100%, there were false-positive and false-negative, and amniocentesis and karyotype analysis were needed for further accurate diagnosis.

### Limitations and Shortcomings

This study is limited to the limitations of time, research level and experimental conditions, and there were many deficiencies to be improved, for example, the cost comparison of traditional methods and NIPT was not included in the research results and the differences between different ethnic groups were not considered, all of which need to be further discussed in the follow-up study.

## CONCLUSION

In conclusion, the average detection rate of non-invasive gene sequencing technology for chromosomal syndrome is above 90%, which is worth promoting and applying in clinic. But it cannot completely replace prenatal screening and diagnosis. According to clinical observation, prenatal screening can screen out high-risk chromosomal diseases and other diseases of pregnant women. Therefore, in order to improve eugenics and eugenics inheritance, this study suggests that prenatal screening and non-invasive gene sequencing technology should be combined with prenatal chromosome karyotype analysis to make a correct diagnosis of fetal birth defects, and mutual cooperation can reduce the blindness of screening fetal birth defects.

### Authors’ Contribution:

**JJB & PJ:** Study design, data collection and analysis.

**XLC & HYZ:** Manuscript preparation, drafting and revising.

**JJB & FXH:** Review and final approval of manuscript.

**JJB** is responsible and accountable for the accuracy and integrity of the work.
